# Dynamic instability of the major urinary protein gene family revealed by genomic and phenotypic comparisons between C57 and 129 strain mice

**DOI:** 10.1186/gb-2008-9-5-r91

**Published:** 2008-05-28

**Authors:** Jonathan M Mudge, Stuart D Armstrong, Karen McLaren, Robert J Beynon, Jane L Hurst, Christine Nicholson, Duncan H Robertson, Laurens G Wilming, Jennifer L Harrow

**Affiliations:** 1 Wellcome Trust Sanger Institute, Wellcome Trust Genome Campus, Hinxton, Cambridge, CB10 1SA, UK; 2 Proteomics and Functional Genomics Group, Department of Veterinary Preclinical Science, University of Liverpool, Crown Street and Brownlow Hill, Liverpool, L69 7ZJ, UK; 3 Mammalian Behavior and Evolution Group, Department of Veterinary Preclinical Science, University of Liverpool, Leahurst, Neston, CH64 7TE, UK

## Abstract

**Background:**

The major urinary proteins (MUPs) of *Mus musculus domesticus *are deposited in urine in large quantities, where they bind and release pheromones and also provide an individual 'recognition signal' via their phenotypic polymorphism. Whilst important information about MUP functionality has been gained in recent years, the gene cluster is poorly studied in terms of structure, genic polymorphism and evolution.

**Results:**

We combine targeted sequencing, manual genome annotation and phylogenetic analysis to compare the *Mup *clusters of C57BL/6J and 129 strains of mice. We describe organizational heterogeneity within both clusters: a central array of cassettes containing *Mup *genes highly similar at the protein level, flanked by regions containing *Mup *genes displaying significantly elevated divergence. Observed genomic rearrangements in all regions have likely been mediated by endogenous retroviral elements. *Mup *loci with coding sequences that differ between the strains are identified - including a gene/pseudogene pair - suggesting that these inbred lineages exhibit variation that exists in wild populations. We have characterized the distinct MUP profiles in the urine of both strains by mass spectrometry. The total MUP phenotype data is reconciled with our genomic sequence data, matching all proteins identified in urine to annotated genes.

**Conclusion:**

Our observations indicate that the MUP phenotypic polymorphism observed in wild populations results from a combination of *Mup *gene turnover coupled with currently unidentified mechanisms regulating gene expression patterns. We propose that the structural heterogeneity described within the cluster reflects functional divergence within the *Mup *gene family.

## Abstract 2

Targeted sequencing, manual genome annotation, phylogenetic analysis and mass spectrometry were used to characterise major urinary proteins (MUPs) and the *Mup* clusters of two strains of inbred mice.

## Background

Communication between conspecifics mediates such interactions as mate choice, parental care and territory defense. Whilst higher primates employ vocalization and visual display for these purposes, many other mammals communicate chiefly by the use of chemical messengers in the form of scent [1]. Human urination performs a purely excretory function; the urine of the house mouse *Mus musculus domesticus*, in contrast, is replete with liver-expressed major urinary proteins (MUPs), encoded by a multigene family (*Mup *genes) on chromosome 4 [2,3]. Notably, the human genome contains a single *Mup *pseudogene [4].

In mice, urinary MUPs are key semiochemicals in several facets of non-overlapping *M. m. domesticus *behavior, including both male to male and male to female interactions [5-13]. MUPs are characterized as an eight stranded beta-barrel structure that encloses a hydrophobic pocket, which in turn binds male specific pheromones 2-*sec*-butyl 4,5-dihydrothiazole (thiazole) and 3,4-dehydro-*exo*-brevicomin (brevicomin) [14-16]. Sequestration of volatile molecules within MUPs delays their evaporation from a scent mark, such that a deposit is detectable for hours as opposed to seconds [17]. In addition to a role in pheromone release, MUPs also communicate information directly. In wild mice, the MUP profile is stable and highly polymorphic: 8 to 14 MUPs are typically detected in each adult individual by electrophoretic separation, with only certain close relatives excreting the same set of molecules [3,9,12,18]. Selective cross-breeding of wild mice and the manipulation of MUP profiles using recombinant molecules have allowed us to conclude that mice remember and distinguish between the profiles of conspecifics; MUPs thus convey an individual recognition signal [6,9,19]. However, certain MUPs are also present in female urine, though at lower concentrations [3,20], and mice avoid inbreeding with very close relatives sharing the same MUP phenotype [12]. Females also preferentially associate with *Mup *heterozygous males [13]. The efficiency of pheromone binding varies dramatically between specific proteins [21,22], suggesting that the gene cluster contains divisions of functionality that are currently uncharacterized. Finally, not all MUPs are excreted in urine, with the transcription of specific *Mup *genes having been detected in mammary, parotid, sublingual, submaxillary and lachrymal glands [22-24]. The function of such non-urinary MUPs is poorly understood, although it is possible to envisage similar communication roles between mother and offspring, delivered through milk, saliva or even tears.

The extreme heterogeneity of the MUP profile in wild mice has only recently been established as most laboratory work has focused on inbred strains, typically C57BL/6J (henceforth B6) from the C57-related strain genealogy and BALB/c from the Castle's mice lineage [25]. The MUP profiles of inbred mice do not vary appreciably between individual adults of the same sex and strain, although the B6 and BALB/c strain profiles are distinct [16]. However, our understanding of the genomic organization of the *Mup *gene cluster lags behind our knowledge of protein functionality, essentially due to complexities in obtaining contiguous genome sequence over the region; the genomic information that has been gleaned was largely generated during the pre-genome sequencing era [26-28]. As such, it is unclear whether the distinct phenotypic profiles of individual mice result from genic polymorphism or variation in gene expression patterns, or perhaps a combination of the two. Little is known about the evolution of the *Mup *gene family, in particular regarding the relationship between urinary MUPs and non-urinary MUPs, and between those MUPs that do and do not exhibit sexually dimorphic expression. It is anticipated that an understanding of the evolution of the *Mup *cluster will, in turn, offer insights into the population dynamics of MUP heterogeneity.

We report here targeted sequencing, detailed annotation and phylogenetic analysis in an in-depth genomic analysis of the *Mup *region of B6 mice. The architecture of the cluster is reconciled with urinary protein expression data, and we propose a functional divergence within the gene family linked to organizational heterogeneity, which in turn reflects differing modes and tempo of evolution. We have also generated a comparable amount of genomic sequence and new protein phenotype data from 129 strain mice. These data allow us to develop a model in which ongoing *Mup *genomic instability facilitates phenotypic variation, and ultimately drives the evolution of mouse behavior.

## Results

### Analysis of B6 and 129S7 genomic sequences

Whilst efforts to close all remaining sequence gaps in the mouse genome are ongoing, a targeted attempt to improve the B6 tile path across the *Mup *cluster was made as part of this study. The selection of bacterial artificial chromosome (BAC) clones from FingerPrinted Contig (FPC) proved to be partially successful [29], with BACs CT572146 and CR550303 added. However, a parallel strategy based around the sequencing of B6 fosmid ends from the WIBR-1 library proved unsuccessful (data not shown). The mapping difficulties result from the high level of sequence conservation within the repeat elements (see below), and they are not unprecedented; many of the remaining euchromatic sequence gaps in both the mouse and human genomes are found within regions containing high-identity sequence repeats, often linked to gene families (unpublished data and [30]). The difficulties faced here are therefore symptomatic of a wider problem in genome sequencing, the solution to which may depend on the further development of new or existing technologies such as optical mapping [31]. At present we do not speculate as to the size of the sequence gaps. The current 'finished' tile path for this region can be viewed in Ensembl as part of the mouse NCBIm36 assembly [32].

**Figure 1:**
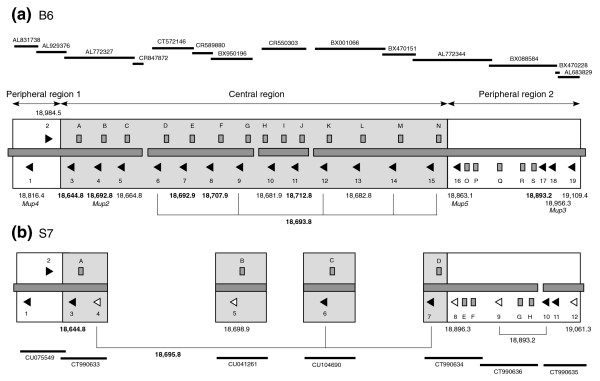
Schematic view of **(a) **B6 and **(b) **S7 *Mup *clusters. The tiling path of BAC clones is indicated by black lines with accession numbers listed. Predicted genes are represented by triangles, pseudogenes by rectangles. Predicted genes are numbered from the 5' direction independently in both strains; official names acquired by certain *Mup*s based on cDNA sequences are listed as appropriate. Pseudogenes are listed alphabetically. Open triangles within the S7 sequence represent gene loci with CDSs that differ from their B6 counterparts, or in the case of gene 5 have no equivalent locus. The gray background shading within the center of each cluster contains those B6 genes and pseudogenes (and S7 equivalents) that form distinct clades within the phylogenetic analysis presented in Figure 2; the loci within the unshaded peripheral regions form isolated nodes. The calculated weight of the mature protein derived from each gene in B6 is indicated, with masses of non-equivalent S7 genes also being listed. Masses that correspond to mass spectrometry peaks identified in Figure 5 are highlighted in bold. The protein corresponding to B6 gene 18 has been identified by other methods (Figure 6); we predict that the calculated mass of the protein does not reflect the urinary mass due to the occurrence of glycosylation (see Results). B6 gene 11 matches closely to an additional protein mass we have previously identified in fractionated urine [21] (see Results). There are three non-equivalent sequence gaps within the central regions of both B6 and S7; the ordering of the central contigs presented here is arbitrary. The S7 genomic sequence includes the Tscot and Zfp37 loci, which flank the cluster in B6 (not shown), indicating that the start and end of the cluster are present. Ignoring gap regions, the B6 cluster is 1.56 Mb in size, the S7 cluster 0.72 Mb.

Figure 1a displays our manual annotation of the *Mup *cluster of the B6 genome, this being done in accordance with the criteria of the Vega genome browser resource [33] (see Materials and methods). There are 19 predicted genes and 18 loci that are pseudogenes (variously due to frameshifts, exon deletions and stop codons). However, the presence of three gap regions within the tiling path indicates that the full complement of *Mup *loci is not yet represented. The aligned protein translations from each predicted functional *Mup *are presented in Additional data file 1. The first approximately 30 amino acids of each MUP is a signal peptide sequence, excised from the mature protein. The following discussions discount this sequence, although observed variation in these signals may have unappreciated roles in, for example, protein localization [34].

**Figure 2:**
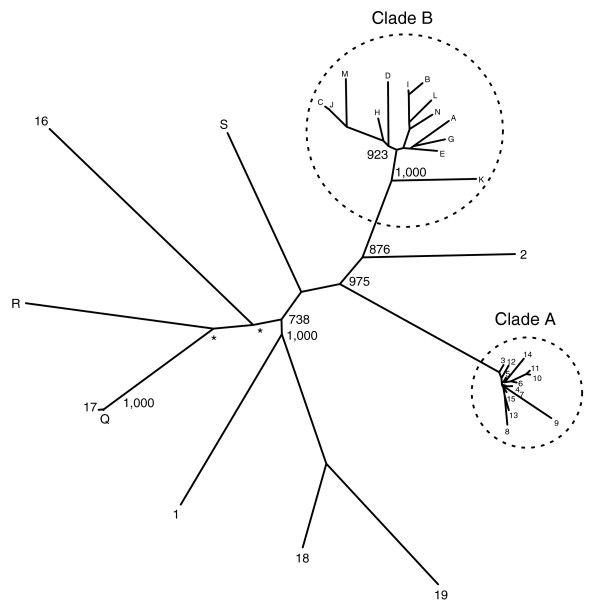
Phylogenetic analysis of B6 *Mup *loci. This unrooted tree was constructed using intron 2, which has an average size of 766.9 bp. Nodes with a bootstrap support of less than 700/1,000 replicates are marked with an asterisk, with the exception of those nodes within the clades marked 'A' and 'B' which are, in general, poorly supported. Numbers and letters at each node refer to genes and pseudogenes, respectively, as annotated in Figure 1a. Pseudogenes O and P are not present as these partial loci do not contain an adequate portion of intron 2; similar phylogenetic analysis with different sequence indicates that these pseudogenes also form isolated nodes (data not shown).

A neighbor-joining tree of B6 *Mup *loci was constructed using intronic sequences (Figure 2). Three points of particular interest stand out. Firstly, the distinct clade marked A consists of the 13 predicted genes that co-localize within the central portion of the cluster (Figure 1a); we refer to these as central loci. Assuming a mouse/rat divergence of 12-24 million years ago (Mya) and an average of 0.166 substitutions per neutral site between loci within the mouse lineage [35], the timing of the oldest duplication event within this clade is predicted at 1.2-2.4 Mya. Secondly, the pseudogenes present within the central region also cluster together (clade B), suggesting their propagation occurred by the serial duplication of an existing pseudogene. Finally, in contrast, the remaining genes and pseudogenes form distinct isolated nodes, and these loci flank the central genes on the periphery of the cluster; we refer to these as peripheral loci. The timing of the oldest divergence amongst the lineage of these genes is estimated at 11.2-22.4 Mya between genes 16 and 19; the timing of the minimum at 4.4-8.8 Mya between genes 18 and 19. The topology of this intronic tree is recapitulated by a phylogenetic analysis based on coding sequence (CDS; data not shown); the central gene CDSs share an average nucleotide identity of 99.2%, and the peripheral genes 88.2%.

**Figure 3:**
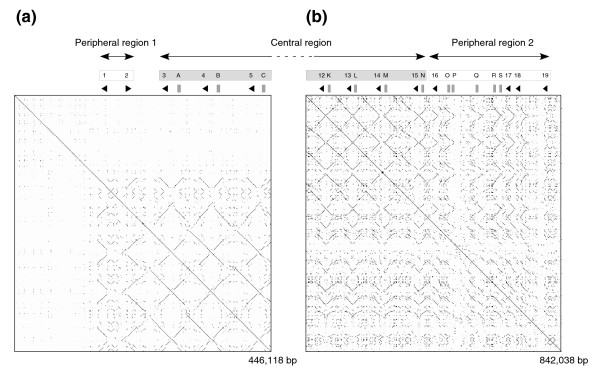
Self comparison of **(a) **B6 proximal contig AL181738 to CR847872 and **(b) **distal contig BX001066 to AL683829. Genes and pseudogenes are annotated as in Figure 1. Loci that form isolated nodes in the phylogeny presented in Figure 2 are boxed in white; those genes and pseudogenes that form respective clades marked A and B in Figure 2 are boxed in gray.

Figure 3 shows dot-plot analyses of the proximal (Figure 3a) and distal (Figure 3b) contigs of the *Mup *cluster. Both contain a transition towards the center of the cluster from a region of low structural definition into a lattice-like array of homogenized sequence. The array comprises the tandem duplication of 14 complete or partial (due to sequence gaps) 80 kb inverted-duplication cassettes, which contain in each complete case a predicted gene and a pseudogene pair corresponding to the loci in clades A and B, respectively, from Figure 2. The average base-pair sequence identity approximates to 98% between cassettes, although sub-regions of alignment are frequently identical over stretches of several kilobases. Dot-plot analysis of the 23 kb of sequence flanking the breakpoint between gene 4 and pseudogene B, which lie on adjacent cassettes, is presented in Additional data file 2. The breakpoint corresponds precisely to the location of a murine endogenous retrovirus (ERV), modified into an inverted duplication. This same sequence conformation is observed between each of the central array cassettes. Provirus elements are known to mediate non-allelic homologous recombination (NAHR); the male-infertility linked AZFa microdeletions on human chromosome Y, for example, are caused by NAHR between HERV15 elements [36]. We thus predict that the central cassette repeat unit was formed by recombination between nearby ERV elements.

We have sequenced and annotated seven BACs from the genome of the 129S7/AB2.2 inbred mouse strain (henceforth S7). The 129 lineage diverged from the C57-related lineage early in the 20th century in a manner that was poorly documented [25]. However, recent investigations have confirmed that the parental line was not inbred before divergence, and subsequent inbreeding of the separated lineages has fixed distinct patterns of wild genetic variation [37]; differing genomic segments of C57, 129 mice and other lineages originate variously from *M. m. musculus*, *M. m. domesticus *and *M. m. castaneus *subspecies [38,39]. It is clear that the essential architecture of the B6 *Mup *cluster is conserved in S7 (Figure 1b). However, five of the twelve S7 gene loci have either amino acid substitutions compared with their corresponding B6 genes or else do not have equivalent loci; these differences are discussed alongside the protein phenotype data below.

### Analysis of B6 and 129S5 phenotypic profiles

**Figure 4:**
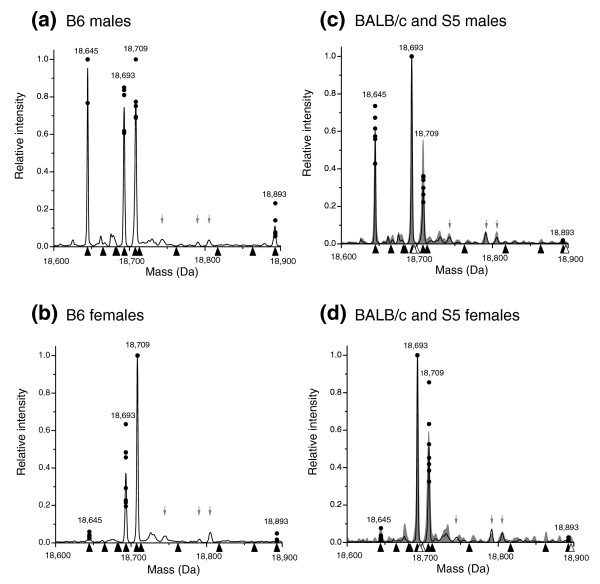
ESI-MS spectra of MUP isoforms in urine samples according to sex and strain. Black lines show average for **(a) **male B6 (n = 5), **(b) **female B6 (n = 8), **(c) **male BALB/c (n = 5), **(d) **female BALB/c (n = 7), and circles show individual values for the relative intensity of each major peak (expressed relative to the base peak, the highest peak in each spectrum, which is set to 1). The mass spectra for a male and female S5 are shown shaded in gray in (c,d), respectively. A duplicate analysis on male and female S5 mice, non-sibling to those above, produced identical results within the boundaries of measurement error. Black arrowheads on the x-axis indicate predicted masses from the B6 genome analysis; unfilled arrowheads additional masses from the S7 genome analysis. Gray arrows above the x-axis indicate known +98 Da adducts of major mass peaks. No consistent peaks were detectable in the range 18,900-19,200 Da (Additional data file 4). The spectra for each individual sample from B6, 129 and BALB/c mice are shown in Additional data file 5.

The protein content of mouse urine is almost exclusively MUPs, expressed at high concentrations. Accordingly, we have developed a phenotypic survey based on electrospray ionization of the protein mixture, generating a complex and overlapping set of multiply charged ions that can be deconvoluted to yield a mass profile of the urinary MUPs. The resolution of this method is ±2 Da, which is inadequate to resolve proteins containing, for example, an Asp/Asn substitution, but which allows many proteins to be unambiguously identified. Although the relative intensities of each peak can be taken as a semi-quantitative index of abundance, we caution against over-interpretation of the profiles in this regard, as MUP expression is subject to developmental and endocrinological control and differences between individuals of the same sex and strain in the relative amounts of individual MUPs are evident (Figure 4).

Figure 4 shows average processed electrospray ionization (ESI) mass spectra derived from the urine of adult male and female B6 mice (Figure 4a,c) and male and female BALB/c mice (Figure 4b,d); these spectra match our previously reported results [16,21]. Previously unreported spectra obtained from two male and two female adult 129S5 (henceforth S5) urinary samples are superimposed onto the BALB/c data. The S5 strain is closely related to S7; the lineages were separated in 1987 from the ancestral inbred 129/Sv stock, with the latter undergoing a mutation in the hypoxanthine guanine phosphoribosyl transferase 1 locus [25]. Neither of these 129 lineages has been outbred with wild mice or crossed with other inbred strains since their separation. Although mice inbreeding programs are designed to minimize genetic drift, this process undoubtedly occurs at low levels both between and within specific lineages [40,41]. We do not, therefore, reject out of hand the possibility that there is minor *Mup *genomic variation either within B6 and BALB/c or between S5 and S7.

The spectra from the S5 males comprised three MUPs, corresponding within 1 Da to the three BALB/c peaks at 18,645 Da, 18,693 Da and 18,709 Da, whilst the spectra from the females of both of the strains contained two MUPs of masses 18,693 Da and 18,709 Da. As well as equivalent peaks at 18,645 Da, 18,709 Da and 18,693 Da, B6 male mice excrete a mass of 18,893 Da not observed in S5 or BALB/c. The genomic annotation of B6 and S7 *Mup *genes presented in Figure 1 allows us to reconcile the urinary MUPs we have identified in Figure 4. This preliminary relationship is summarized below. We have also examined the transcriptional profile of these loci by comparison against the GenBank sequence database [42].

#### Observed mass 18,645 Da

MUPs of this mass, observed in all three strains, match to the calculated mass of 18,644.8 Da for B6 and S7 gene 3, which have identical translations. The protein is predominantly expressed in males, but there is some evidence of low expression (typically <5%) in females (Figure 4b,d). There is extensive transcriptional support for this locus from liver-derived libraries of B6, FVB/N (a distinct lineage from 129/BALB/c and C57 mice [25]) and BALB/c strains.

#### Observed mass 18,709 Da

MUPs of this mass, observed in both sexes of all three strains, are matched to gene 8 in B6 (18,707.9 Da), which lacks transcriptional support from any strain. There is no corresponding gene in the S7 genomic sequence at present; we predict this locus resides within a gap region.

#### Observed mass 18,693 Da

MUPs of this mass, identified in both sexes of all three strains, can be matched to seven of the central array genes of B6 (4, 6, 7, 9, 12, 14, 15), five of which (6, 9, 12, 14, 15) have identical translations except for their signal peptides (Additional data file 1). Genes 4 and 7 have predicted masses that differ by less than 1 Da from both each other and that of the five identical translations; such proteins are indistinguishable at the intact protein level by the analysis conducted here. However, in previous work combining ESI mass spectrometry (ESI-MS) with anion exchange chromatography we observed that this 18,693 spectral peak in BALB/c actually consists of two MUP species that can be separated by their charge; we thus now predict that these distinct proteins are derived from central array genes that differ by one or few amino acid substitutions [16]. We did not find evidence for the similar excretion of charge variants in B6. However, we characterized individual anion exchange fractions from B6 urine and identified a protein mass at 18,713 that had co-eluted with the 18,693 Da material [21]; we now link this protein mass to B6 gene 11 (calculated mass 18,712.8 Da). Mass 18,693 corresponds to S7 genes 4, 6, and 7. The majority of gene loci from both B6 and S7 are supported by transcriptional evidence, invariably from liver-derived libraries. Note that B6 gene 4 and S7 gene 4 differ by a single amino acid substitution: a Gln/Glu change at position 13 (Additional data file 1); the S7 gene 4 has a translation identical to that of B6 genes 6, 9, 12, 14 and 15.

#### Observed mass 18,893 Da

MUPs of mass 18,893 Da correspond to gene 17 in B6 (18,893.2 Da); the protein is predominantly expressed in B6 males (Figure 4a,b) and is thus sexually dimorphic. This locus is supported by cDNA Em:BC089613, derived from B6 male liver, and Em:BC092096, derived from FVB/N male liver. The absence of this protein was previously noted in the urine of 6 out of 84 male wild mice [21], and in this report the protein mass is undetected in the urine of S5 and BALB/c mice of both sexes. This S5 result was surprising, since S7 gene 10 has an identical CDS to B6 gene 17 (Additional data file 1). Also, S7 gene 9 is equivalent in location to B6 pseudogene Q, yet this locus has a CDS identical to that of B6 gene 17/S7 gene 10. We further investigated the relationship between these four 18,893-associated loci in order to explain this non-conformity.

B6 pseudogene Q is classified as such due to the loss of 20 bp of sequence within exon 4; this deletion has been confirmed by checking the original whole-genome shotgun data across this region [43]. A dot-plot comparison of the two 18,893-associated B6 duplication regions is displayed in Additional data file 3. Ignoring the presence of a unique IAPLTR-1 retrotransposon within pseudogene Q, it is clear that the loci were duplicated as part of a larger event involving 29 kb of sequence. The proximal breakpoint occurs within the solitary long terminal repeat of an IAPLTR2 element, whilst the downstream breakpoint occurs within an ERV element homologous to those associated with the central array duplications (Additional data file 2). Over the proximal 22 kb the nucleotide identity between the two regions averages as 99.6%; after this point the similarity drops abruptly, averaging at 92.2% over the final 7 kb. The point of transition occurs 795 bp in the 5' direction from the transcriptional start site, and it does not correspond to any known transposable elements or repetitive sequence. This pattern of nucleotide identity could be explained by the occurrence of a 22 kb duplication on top of the site of a pre-existing duplication event. The genomic sequence of S7 contains the same duplication architecture (not shown).

**Figure 5:**
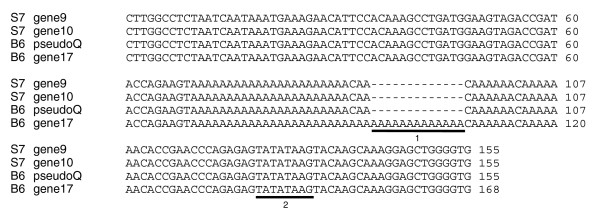
Alignment of promoter regions of mass 18,893-associated loci. The alignment ends immediately prior to the predicted common transcriptional start site of *Mup *loci. The first underlined region indicates a C/A substitution followed by an additional 13 A residues present in B6 gene 17. Similar though non-identical A-rich regions are found in the equivalent location at each *Mup *loci. The second underlined region is the TATA-box sequence, common to all *Mup *loci.

We examined the DNA sequence up to 5 kb upstream of these four loci in order to identify changes to potential promoter elements. Figure 5 displays a portion of the alignment of the sequences immediately upstream of the transcriptional start site. The functional B6 gene 17 contains one notable difference: the presence of an extra 13 [A]s and a nearby C/A substitution within an A-rich site 30 bp upstream of the TATA-box element. We observe that similar (though non-identical) A-rich sites are present in the same location at each predicted *Mup *locus, and their presence and putative functionality have previously been highlighted in the equivalent rat gene family [44,45]. These elements do not appear to be protein binding sites. Instead, they may act as spacer elements, affecting transcriptional efficiency by adjusting the distance between the TATA-box and upstream control elements.

#### Protein corresponding to B6 gene 18

**Figure 6:**
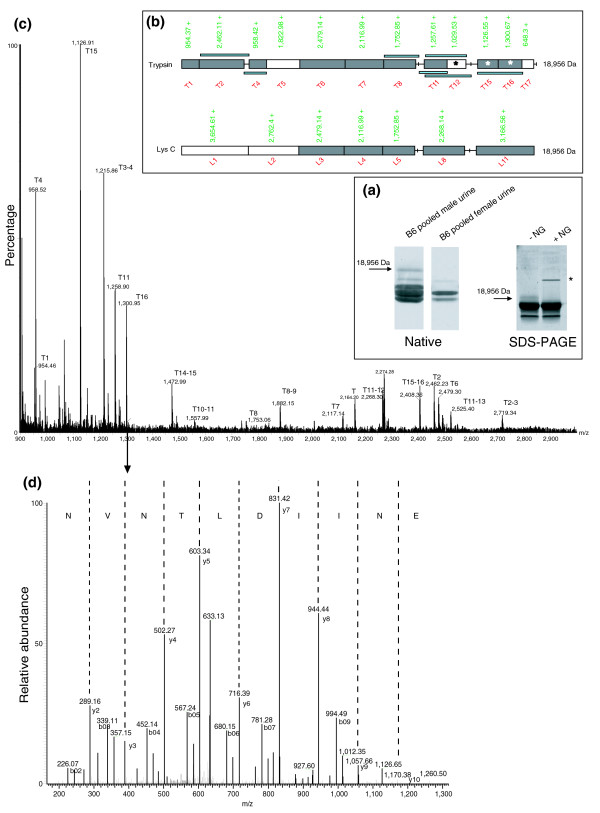
Identification of 18,956 Da MUP by gel electrophoresis, tandem mass spectrometry and peptide mass fingerprinting (PMF). **(a) **Urine pooled from five B6 males and five females was first resolved by non-denaturing (native) or SDS-PAGE electrophoresis (8 μg protein loaded). The male specific band indicated by the arrow was excised from the gel and digested with trypsin or endopeptidase LysC for peptide mass fingerprinting. **(b) **The peptide maps define peptides (trypsin: T1...T17, endopeptidase LysC: L1...L11) that would be obtained from the MUP of unmodified mass 18,956. Peptides that were identified by PMF (shown in **(c)**) or by MS/MS (shown in **(d)**) are shaded or highlighted with an asterisk. Overlaid narrow bands define peptides identified as part of a missed cleavage. (c) A representative MS/MS spectra of peptide ENIIDLTNVNR, m/z 1,300.67, [M+2H]^2+ ^650.7. This protein contains a putative glycosylation site at Asn66 (AFVENITVLENSLVFK77, peptide T5) and, after digestion with N-glycanase (NG, enzyme band indicated by an asterisk), shifted in electrophoretic mobility (a).

B6 gene 18/S7 gene 11 has extensive support for liver transcription from both B6 and FVB/N mice, although the calculated mass of 18,956.3 Da is not observed by mass spectrometry. The inability to observe this mass probably stems from the fact that the sequence contains a potential N-linked glycosylation site at Asn_66 _(62 AFVENITVLENSLVFK77, tryptic peptide T5) that would, if modified, increase the mass. However, we have isolated and identified this protein in male B6 urine using a combination of gel electrophoresis, tandem mass spectrometry and peptide mass fingerprinting (Figure 6). A minor protein species is evident in native gel electrophoresis as a low mobility band, and on SDS-PAGE as a higher mass band (Figure 6a). In both instances, the bands could be excised and digested with trypsin or endopeptidase LysC, generating comprehensive peptide mass fingerprints that permitted unambiguous identification of the protein as that encoded by gene 18. Tandem mass spectrometry of fragment ions allowed recovery of peptide sequence data; representative data for one such tryptic fragment (T16: ENIIDLTNVNR, m/z 1,300.7) confirm unambiguously the identity of this protein. Confirmation of the glycosylation status was provided by treatment with endoglycosaminidase, after which the low mobility/high mass band on SDS-PAGE disappeared, consistent with it being glycosylated. Although present at a comparatively low concentration in urine (less than 2% of total urinary MUP protein), this peripheral MUP appears to be sexually dimorphic since it cannot be detected in female urine. We have not yet examined the presence of the equivalent gene product in 129 or BALB/c mice.

### Eight *Mup *loci lack corresponding mass spectrometry data

There are eight predicted genes across B6 and S7 that do not correspond to mass spectrometry peaks in either strain (or have corresponding proteins identified through our other methodologies detailed above). Interestingly, three of these genes do have predicted masses for which we have readily detected closely matching spectra of 18,666 and 18,682 in a parallel analysis on wild-derived *M. m. domesticus *mice (unpublished data and [46]). These are B6 central array genes 5 (calculated weight 18,664.8 Da), 10 (18,681.9 Da), and 13 (18,682.8 Da), each of which lacks transcriptional support. This suggests that these loci are not active at detectable levels in B6 or S7, but are in certain wild individuals. In contrast, we have never observed a protein mass corresponding to S7 gene 5 (predicted 18,698.9 Da), which has two amino acid substitutions not present in any B6 loci: Asp/Val at position 34 and Ser/Arg at position 128 (Additional data file 1). However, both of these positions are variant in other B6 urinary proteins with alternative substitutions, raising the possibility that the two sites display functional polymorphism. This S7 gene is supported by a single EST, Em:BI256026, derived from FVB/N liver.

Strikingly, the four remaining genes make up six of the genes located within the peripheral regions of both strains. Two of these genes have previously been described as expressing non-urinary MUPs. Transcription of the B6 gene 1/S7 gene 1 has been described in lachrymal and parotid gland tissue [23], and the set of cDNAs and ESTs corresponding to this locus in GenBank are limited to these tissues. The second is B6 gene 16/S7 gene 8, for which the S7 and B6 CDS differ in three amino acid positions. The S7 form of the locus is identical to BALB/c cDNA Em:M16360, a major transcript in the submaxillary gland [24]; again, there is no liver transcriptional support in GenBank. This is the only MUP to lack a tyrosine residue at position 121 within the internal binding cavity of the protein. This residue may have a direct role in ligand binding [22,47], raising the possibility that the submaxillary protein might have profoundly altered ligand specificity, or may operate in the absence of bound ligand.

The functional status of the two remaining loci is unclear. B6 gene 2/S7 gene 2 has two associated ESTs, Em:CF894970 and Em:AV585390, derived from distinct undifferentiated embryo stem cell libraries, although the protein has never been identified experimentally. Finally, B6 gene 19/S7 gene 12, which differ in one amino acid position, lack the non-coding final exon of other *Mup *genes, suggesting they may be pseudogenes in spite of their intact CDS. However, FVB/N liver ESTs Em:BI146097 and Em:CA478551 indicate the locus is transcribed in this strain at least, although again there is no evidence for secretion of the protein.

## Discussion

**Figure 7:**
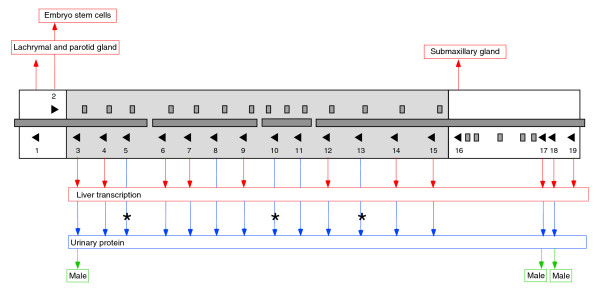
Summary of *Mup *cluster transcriptional and phenotypic profiles. The cluster of B6 is presented as annotated in Figure 1. Red arrows and red text boxes indicate tissues in which transcription can be confirmed for each locus based on the presence of 100% supporting cDNAs or ESTs in GenBank [42] (whilst allowing for poor-quality sequence at the immediate 5' and 3' ends for ESTs); blue arrows and blue text boxes indicate genes where a corresponding protein has been detected in urine. The asterisks marking genes 5, 10 and 13 indicate that potentially corresponding proteins for these loci have previously been detected in wild mice, but not in the inbred mice studied in this investigation [46] (see Results). Genes 4, 6, 7, 9, 12, 14 and 15 each have proteins with predicted masses that can be matched to detected mass 18,693 Da; it is currently unclear which of these loci contribute to the protein peak in Figure 4. Proteins that exhibit sexual dimorphism (being detected in males but not females) are indicated by green arrows and text boxes marked 'Male'.

This is the first in depth analysis of the *Mup *gene clusters of two distinct strains of mice, strengthened by resolution of the distinct urinary profiles of these mice alongside their respective gene complements. We have linked our experimental observations to a combination of structural and phylogenetic analyses of the cluster, and observe that the region contains a distinct pattern of organization, with the central and peripheral sections being structurally and phylogenetically distinct. This appears to reflect differing modes of evolution, which may be linked to a division of functionality within the cluster. Figure 7 summarizes the total information now available regarding the transcriptional and phenotypic profile of the *Mup *gene cluster. It must be reiterated that our investigation has studied inbred laboratory mice and not wild mice. Heterozygous wild males typically contain approximately twice as many MUPs in urine as inbred males, and it seems a fair assumption that this increase is due at least in part to heterozygosity across the cluster [3,9,12,18]. It should also be noted that the human selection of mouse breeding pairs in the development of laboratory strains over the last hundred years may have imparted a degree of artificial selection on the *Mup *clusters, given that these genes directly influence various aspects of mouse behavior.

### The central region is likely subject to concerted evolution

The genes within the central array of both B6 and S7 differ by an average of just 0.8 bp within their CDS, and since an almost identical degree of nucleotide identity is maintained across their intronic sequence, this similarity cannot be due to purifying selection alone. Instead, the homogenized nature of the central array indicates the action of concerted evolution [48], which operates via both NAHR and gene conversion events. The action of concerted evolution is typically demonstrated by comparing the alignment of paralogs from a variety of species [49]. Here, ambiguities arising from the incomplete nature of the B6 and S7 genomic sequences limit the value of a detailed analysis at present. However, the alignment of central B6 MUP proteins 3, 5, 7, 10, 11 and 13, displays mosaicism in the pattern of amino acid substitutions, indicative of recombination (Additional data file 1). We predict NAHR generates novel *Mup *genes by recombining mutations from different cassettes, whilst the action of gene conversion generates polymorphism where single conversion events between *Mup *genes result in only partial homogenization [49-51]. By this model NAHR also causes the continuing expansion and contraction of the cluster via duplication and deletion events, potentially involving multiple cassettes; a recent report into the incidence of murine global copy number variation highlighted the *Mup *cluster as varying dramatically in size between different inbred strains [52]. We predict that the central *Mup *region is polymorphic in wild populations, both in terms of copy number and in the specific loci that are present, and that the ongoing action of concerted evolution acts to prevent the long-term differentiation of these *Mup *genes [53].

### The central region may confer self/non-self recognition

MUPs confer a 'signature' of individuality and kinship identity via the highly polymorphic set of proteins excreted by wild individuals [3,6,12]. The central array of *Mup *genes could generate the requisite genetic polymorphism for this functionality by the ongoing action of point mutation and concerted evolution. This individuality coding may overlap with functionality in pheromone binding; interestingly, however, our earlier work showed that the central 18,645 Da, 18,709 Da and 18,693 Da proteins are poor binders of thiazole compared to the peripheral 18,893 Da protein [21] (see below). Furthermore, individuality coding appears to function in both male and female mice, and it is noteworthy that the two MUP peaks identified in the female mice of the three strains studied here correspond to central genes.

Our analysis indicates that the oldest array duplication events occurred 1.2-2.4 Mya, although this value may be an underestimate due to gene conversion. As it stands, the timing for the expansion of the central array overlaps with the separation of *M. m. domesticus *from the Algerian mouse *Mus spretus *and eastern European mouse *Mus macedonicus *lineages around 1.5 Mya [54,55]. Wild *M. m. domesticus *mice have higher population densities than *M. spretus *and *M. macedonicus*, and it is believed that the former became commensal alongside the development of human civilization 10,000 years ago [56]. We have recently performed mass spectrometry analyses on wild *M. macedonicus *mice captured from different locations, and observed in each case the same solitary mass peak, indicative of a single protein species [46]. Similar analyses of five wild-caught *M. spretus *males revealed nearly identical individual profiles, in this case consisting of three peaks [57]. We proposed that MUP family expansion and polymorphism arose in *M. m. domesticus *to match a demand for elaborate communication coupled to an increase in social complexity, and we now predict that this acquisition of functionality may have been facilitated by the expansion of the central *Mup *cluster.

### The peripheral regions are likely subject to birth-and-death evolution

The six genes that flank the central array (Figure 1) form isolated nodes in the tree in Figure 2, and do not exist within homogenized cassettes as seen in Figure 3. The divergence of these loci pre-dates the predicted divergence of the *M. m. domesticus*, *M. spretus *and *M. macedonicus *lineages [54,55], falling closer to the estimated divergence of the mouse/rat lineages 12-24 Mya [35]. The exception is the duplication event forming the B6 gene 17/pseudogene Q pair, which represents a much more recent event. Thus, whilst we infer the peripheral regions have a higher degree of structural stability than the central region, the potential for instability persists. There are four other pseudogenes in the downstream peripheral region, which do not represent serial duplications. These observations suggest that birth-and-death evolution is the dominant mode of change over these regions [53,58], whereby gene copies are created by duplication and either acquire functionality or else undergo pseudogenization. By this model, the differences between the CDS of these loci may indicate the acquisition of individualized functions in the absence of homogenization by NAHR. Of the six peripheral MUP proteins, two appear to be predominantly expressed by males in urine (B6 genes 17 and 18), two are non-urinary (1 and 16) and two have never been detected experimentally (2 and 19; Figure 7).

It has been suggested that signaling ligands may be transferred from sequestering urinary MUPs to MUPs in the nasal cavity when a scent mark is investigated [59]. We have previously shown that the 18,893 Da protein sequesters 40% of the total quantity of thiazole in B6 urine, in spite of the significantly lower concentration of this protein compared with the other urinary MUPs [21]. Interestingly, the nasally excreted B6 gene 1 also has a significantly elevated binding affinity for this ligand [21,22]. It is thus noteworthy that thiazole is a male-specific ligand, and that the 18,893 Da protein is normally detected only in male urine; we now suggest that these peripheral MUPs may have evolved in tandem to facilitate thiazole transfer in a sexually dimorphic manner. The 18,893 mass is undetected in the urine of a minority of male wild mice [21], although it remains to be seen whether this results from the loss of this *Mup *gene, amino acid substitutions that affect mass but not functionality, or cryptic regulatory changes (see below). Finally, if our prediction is correct, one may expect that thiazole transfer is compromised in S5 and BALB/c mice, given that these strains lack the 18,893 mass peak; this possibility has yet to be investigated. At present we do not propose a function for the urinary MUP encoded by B6 gene 18 (Figure 6), although it should be recalled that male urine contains ligands other than thiazole, most notably brevicomin [14,15].

### Certain *Mup *genes appear non-transcribed

The S5 protein profile lacks mass 18,893 Da (Figure 4), contrary to our predictions based on the S7 genomic sequence, and this mass is also absent in BALB/c. Additionally, there are three intact CDSs within the B6 central array that match mass peaks previously observed solely in wild-caught mice [46] (B6 genes 5, 10 and 13; Figure 7). Four MUPs thus appear polymorphic at the phenotypic level in a manner that is not coupled to polymorphism at the genomic level in an obvious way. However, genic polymorphism is not limited to the CDS. *Mup *gene regulation is complex, and trans-acting factors known to modulate *Mup *loci (or the equivalent rat loci) include growth hormone [60], thyroid hormone [61], glucocorticoids [62] and androgens [63]. Certain genes lacking protein support may contain cryptic promoter or binding site mutations that impede transcription, and we have identified an intriguing difference in a putative functional element unique to the functional B6 gene 17 (Figure 5). It is also noteworthy that evidence exists indicating *Mup *genes are subject to methylation, albeit in a manner that is currently not understood [64-66].

## Conclusion

Our combination of genome sequencing, annotation and experimental analysis provides a valuable resource for future studies into both the functionality and evolution of the gene family; attempts, in short, to trace the path from *Mup *genotype to MUP phenotype, and ultimately to mouse behavior. We predict that differing modes of evolution within the central and peripheral regions of the cluster reflect functional divergence, with the ongoing occurrence of recombination within the central cluster generating a rapid turnover of polymorphic gene variants, whilst the peripheral loci acquire specialized functions by divergent evolution. These propositions will be tested by the future generation of genomic sequence from other *M. m. domesticus *mice, mice of other species/subspecies, and perhaps other rodents. However, even considering the urinary MUPs alone, it is clear that the link between genotype and phenotype is not straightforward. We predict that the differing MUP profiles of wild mice result from a combination of the set of *Mup *loci a particular genome contains coupled with variation in gene expression patterns. Future progress in genotype/phenotype correlation is thus likely to coincide with an increase in our understanding of *Mup *gene regulation.

## Materials and methods

### Animals and sampling

S5 (129/SvEvBrd) mice were housed at the Wellcome Trust Sanger Institute under standard conditions. Urine from male and female adult mice was collected by bladder massage. BALB/c and B6 mice were housed in the Faculty of Veterinary Science at the University of Liverpool under equivalent conditions, with urine obtained by M Thom in the same manner.

### Clone selection from C57BL/6J and 129S7 libraries

Candidate B6 BACs were selected from the FPC clone library in line with standard procedures [29]. A tiling path of candidate BAC clones from the S7 library was selected from Mouse Ensembl [32] to cover the region of interest in the B6 assembly (NCBI m34) using the BAC end sequence alignment track [37]. Candidate BAC clones were analyzed using *Hin*dIII restriction fingerprinting and assembled into contigs in FPC [29] to allow selection of a minimal tiling path. Both the repetitive nature of the region and the remaining gaps in the B6 tiling path prevented contiguous coverage from the S7 library (see Results).

### Sequencing of C57BL/6J and 129S7 clones

Previous sequencing of the *Mup *region of B6 revealed a high level of repeat within the clones. Therefore, each selected S7 BAC clone had 2 pUC19 subclone libraries prepared with insert sizes of 4-6 kb and 6-9 kb, this combination of insert sizes having proved expedient for manual finishing of B6 clones. Of the seven clones finished, two utilized this combination of sequence data from both subclone libraries. Both lie in the central region, which contains an elevated repeat content (see Results). All pUC subclones utilized were sequenced with AB Big Dye Terminator Mix v3.1™ and the data analyzed on AB 3730 automated sequencing instruments at Hinxton, UK. The data were assembled and subjected to automated primer walking, prior to re-assembly using PHRAP (P Green) and then passed into directed manual finishing for completion to phase 3. The sequences of all BACs generated in this study have been deposited in GenBank [42].

### Annotation and phylogenetic analysis of genomic sequence

Genomic sequence from both strains was analyzed as part of the VEGA project [33]. This involves fully manual gene annotation based on transcriptional evidence; full criteria are detailed on the website. Manual annotation is desirable over automated gene building methodologies when describing homogenous gene clusters. Prior to this analysis the Ensembl gene build contained numerous chimeric *Mup *loci that erroneously spliced together exons from adjacent genes; the Ensembl and Vega gene builds have since been merged [67]. Annotation and dot-plot analyses were performed using in-house software (J Gilbert). Molecular weights of predicted proteins (minus signal peptides) were calculated using the Compute Pi/Mw tool on the ExPASy server [68]. MUPs contain a disulphide bridge between a pair of cysteine residues conserved in all proteins; 2 Da was deducted from each predicted mass to take this modification into account [69]. Repeats were identified using RepeatMasker [70], and further characterized as appropriate using the RepBase resources [71]. For phylogenetic analysis the sequence of intron 2 of each locus for which this sequence was available was excised and aligned using ClustalW [72] followed by manual re-alignment where required. Further analysis was performed using the Phylip software suite [73], using the neighbor-joining methodology with the Kimura-2-parameter model, alongside 1,000 bootstrap replicates.

### MUP preparation

MUPs were purified from urine by spun-column gel permeation chromatography. Columns (5 ml) were packed with pre-swollen Sephadex-G25 that was subsequently equilibrated in deionized water. Excess water was removed from the columns with a 200 g spin for 2 minutes. An aliquot (200 μl) of urine was then loaded onto each column, which was spun for a further 2 minutes at 200 g. The eluent from the column was captured in 1.5 ml polypropylene test tubes and either submitted immediately for analysis or stored at -20°C. Desalted, unfractionated MUPs were diluted 1:500 with 50% (v/v) acetonitrile/0.1% (v/v) formic acid, and desalted ion-exchange fractions were diluted 1:100 with the same diluent prior to mass spectrometry.

### Electrospray ionization mass spectrometry

ESI-MS and tandem mass spectrometry (ESI-MSMS) were performed on a Micromass Q-ToF Micro instrument, fitted with a nanospray source at Liverpool, UK. The electrospray was created from a silver coated glass capillary with a 10 μm orifice (New Objective, Woburn, MA, USA), held at a potential of +2,000 V relative to the sample cone. For measurement of the mass of intact MUPs from B6, BALB/c and S5 mouse urine, a desalted sample was diluted 1:500 with a solution of 50% (v/v) acetonitrile/0.1% (v/v) formic acid and introduced into the mass spectrometer by syringe pump infusion (Harvard Instruments Ltd, Edenbridge, UK) at a rate of 0.5 μl/minute. In this case, the instrument was operated in TOF only mode, with the quadrupole analyzer operating in Rf only mode to allow transmission of all ions. Raw data were gathered between 700 and 1,400 Th at a scan/interscan speed of 2.4/0.1 s. These raw data were subsequently de-convoluted and transformed to a true mass scale using the MaxEnt 1 module contained within the MassLynx 4.0 software, published by the Waters Corporation, Milford, MA, USA. To create the MaxEnt damage model, peak width and resolution parameters of 0.75 Da and 1 Da/channel were used, respectively, and data were processed over the mass range 18,400-19,000 Da. For replicate analysis, true mass spectra were normalized to the most abundant protein and aligned using SpecAlign [74]. The average spectrum was calculated for each strain and sex, together with the mean ± standard error of the mean relative peak height for each mass.

### Native PAGE

Native PAGE was performed essentially as described by UK Laemmli [75]. However, no SDS or reducing agents were included in any of the gel, running or sample buffers. Pooled urine samples were mixed 1:1 with sample buffer before loading. The gel acrylamide concentration was 20%. Electrophoresis was performed at a constant 200 V for 2 h. Protein bands were visualized with Coomassie brilliant blue. Loading increasing volumes of urine does not change the MUP protein banding pattern at the qualitative level (Additional data file 6).

### Deglycosylation

B6 male MUPs were subjected to N-linked oligosaccharide digestion according to the denaturing protocol for the enzymatic deglycosylation kit (Glyko ProZyme, San Leandro, CA, USA). Briefly, 20 μg of protein was diluted in 30 μl of water, 10 μl of 5× incubation buffer, and 2.5 μl of denaturation solution; heated for 5 minutes at 100°C; and cooled to room temperature. Then, 2.5 μl of the detergent solution was added to the sample, which was then digested with 1 μl of N-glycanase for 3 h at 37°C. Protein band mass shifts due to oligosaccharide cleavage were monitored using SDS-PAGE.

### In-gel enzyme digestion

Plugs were removed from protein bands on the native PAGE gel using a thin glass pipette and placed into microcentrifuge tubes. Each gel plug was destained using 100 μl of 50 mM ammonium bicarbonate, 50% (v/v) acetonitrile (trypsin) or 100 μl 25 mM Tris HCl pH 8.5, 50% (v/v) acetonitrile (lys C), and was incubated at 37°C for 30 minutes. This step was repeated until no stain was visible. The plugs were then washed twice in 100 μl 50 mM ammonium bicarbonate (trypsin) or 100 μl 25 mM Tris HCl pH 8.5 (lys C), which was then discarded. The plugs were then incubated with 50 μl of a 10 mM dithiothreitol stock solution. After 30 minutes at 37°C the dithiothreitol was discarded and 50 μl of a 55 mM iodoacetamide stock solution was added to each tube and incubated for 1 h at room temperature in the dark. The iodoacetamide was discarded and the plugs washed twice as above. The plugs were dehydrated in 100% acetonitrile. The plug was rehydrated in 19 μl of 25 mM Tris/HCl, 1 mM EDTA, pH 8.5 (lys C), or 50 mM ammonium bicarbonate (trypsin). Sequencing grade endoproteinase lys-C or trypsin (1 μl, 0.1 μg/μl, Roche, Basel, Switzerland) was added and the digest was incubated overnight at 37°C. The reaction was stopped with 1 μl formic acid.

### Peptide mass fingerprinting

The peptides were analyzed on a matrix-assisted laser desorption ionization time of flight mass spectrometer (MALDI-TOF/MS) (Micromass) operated in reflectron mode with positive ion detection. External mass calibration was determined using a mixture of des-Arg bradykinin, neurotenin, ACTH, and insulin b chain (50 mM each in 50% acetonittrile, 0.1% trifluroactic acid). Samples were mixed 1:1 (v/v) with a saturated solution of α cyano-4-hydroxycinnamic acid in acetonitrile:water:trifluroacetic acid (50:49:1, v/v/v). Peptide mass fingerprints were searched against the MSDB database (release 20063108) using the MASCOT search engine [76]. Peptide mass fingerprint search parameters were set to tolerate a maximum of one missed enzyme cleavage with carbamidomethyl cysteine as a fixed modification and methionine oxidation as a variable modification; peptide mass tolerance was ±250 ppm.

### Tandem MS/MS

In-gel trypsin digests were analyzed using a LTQ ion trap mass spectrometer (Thermo Electron, Hemel Hempstead, UK) coupled online to a L3000 nanoflow HPLC (Dionex, Sunnyvale, CA, USA) equipped with a LC packings PepMap 100 C18 reverse phase column (75 μm internal diameter × 15 cm length, 3 μm particle size, 100 Å pore size). Tandem MS spectra were submitted to MASCOT to search MSDB [75]. MS/MS ion search parameters were set to tolerate a maximum of one missed enzyme cleavage with carbamidomethyl cysteine as a fixed modification, methionine oxidation as a variable modification; peptide mass tolerance was ±1.2 Da, and fragment mass tolerance was ±0.6 Da.

## Abbreviations

B6, C57BL/6J; BAC, bacterial artificial chromosome; brevicomin, 3,4-dehydro-*exo*-brevicomin; CDS, coding sequence; ERV, endogenous retrovirus; ESI, electrospray ionization; FPC, FingerPrinted Contig; MS, mass spectrometry; MUP, major urinary protein; Mya, million years ago; NAHR, non-allelic homologous recombination; S5, 129S5; S7, 129S7; thiazole, 2-*sec*-butyl-4,5-dihydrothiazole.

## Authors' contributions

JMM carried out the genomic and phylogenetic analyses and wrote the manuscript. KM and CN coordinated the sequencing efforts. SDA and DHR performed the protein characterization. RJB and JLH directed the phenotype analyses, analyzed the phenotype data and co-wrote the manuscript. LGW and JLH provided guidance and support in the conception of the genomic analyses. All authors read and approved the final manuscript.

## Additional data files

The following additional data are available. Additional data file 1 is an alignment of the B6 and S7 MUPs. Additional data file 2 is a dot-plot self comparison of genomic sequence between B6 gene 4 and pseudogene B, with the point of alignment inversion seen to correspond to the location of a murine ERV. Additional data file 3 is a dot-plot comparison of the mass 18,893-associated duplication from the B6 genome. Additional data file 4 details the absence of MUP isoforms in the upper mass range of ESI-MS spectra of inbred mouse urine samples. Additional data file 5 details individual variation in ESI-MS mass spectra of MUP isoforms in urine. Additional data file 6 shows that increasing the volume of urine loaded onto a native PAGE gel does not change the banding pattern observed once the essential banding pattern has become visible.

## Supplementary Material

Additional data file 1 Alignment of the B6 and S7 MUPs. Click here for file

Additional data file 2 The point of alignment inversion is seen to correspond to the location of a murine ERV. Click here for file

Additional data file 3 Dot-plot comparison of the mass 18,893-associated duplication from the B6 genome. Click here for file

Additional data file 4 Details of the absence of MUP isoforms in the upper mass range of ESI-MS spectra of inbred mouse urine samples. Click here for file

Additional data file 5 Individual variation in ESI-MS mass spectra of MUP isoforms in urine. Click here for file

Additional data file 6 Increasing the volume of urine loaded onto a native PAGE gel does not change the banding pattern observed once the essential banding pattern has become visible. Click here for file
