# Supplementary material for: Dynamic instability of the major urinary protein gene family revealed by genomic and phenotypic comparisons between C57 and 129 strain mice
Source: Genome Biol. 2008 May 28;9(5):R91. doi: 10.1186/gb-2008-9-5-r91 (PMC2441477; doi:10.1186/gb-2008-9-5-r91)
Supplement: Additional data file 1 — Alignment of the B6 and S7 MUPs. [file gb-2008-9-5-r91-S1.pdf]

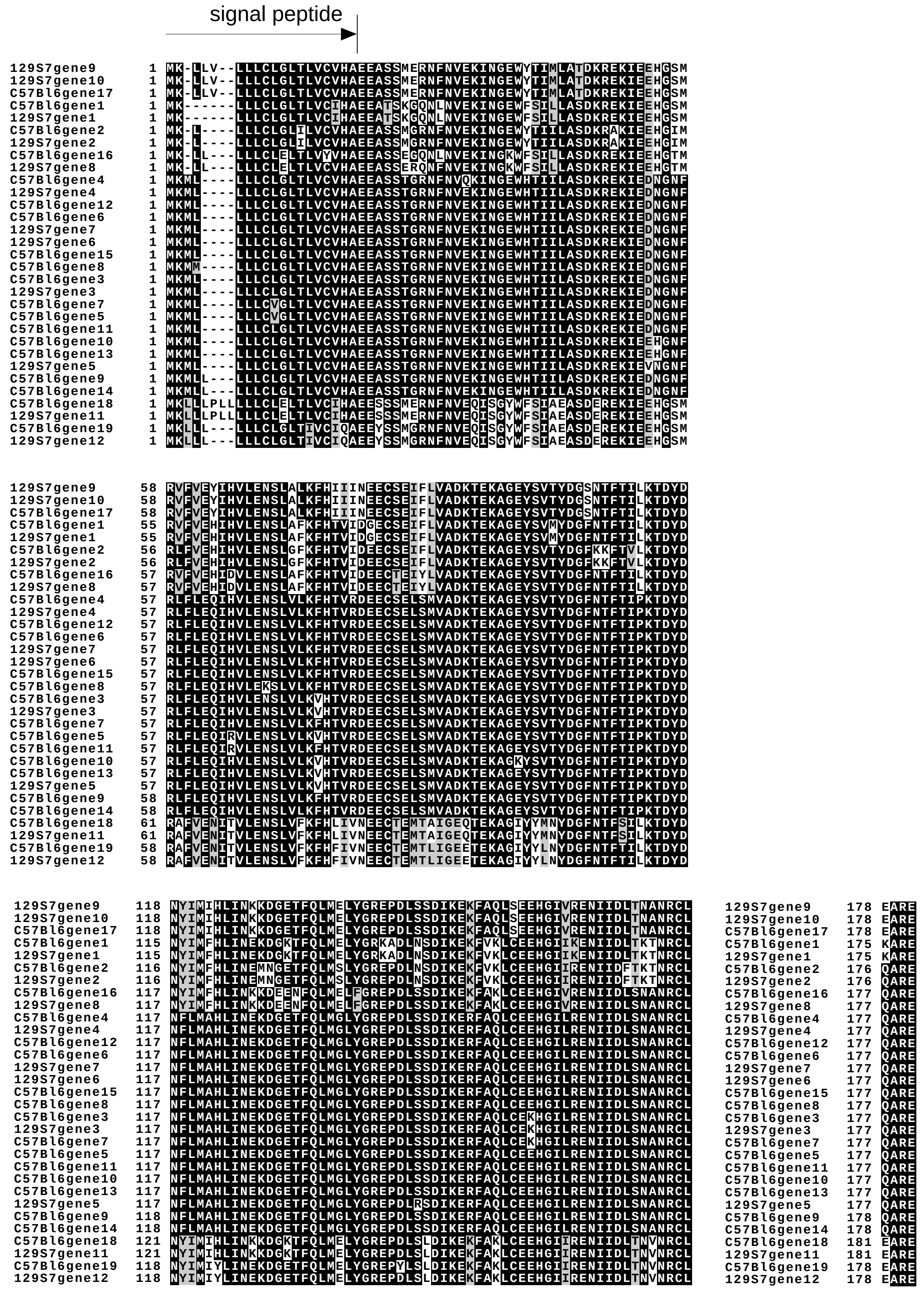

Additional data file 1. Alignment of the B6 and S7 MUPs. Each predicted coding sequence from both strains as annotated in Figure 1 is included. The signal peptide common to all MUPs is indicated; note that certain MUPs are identical over the mature peptide sequence whilst differing from one another in their signal peptide. All amino acid positions in the text are numbered relative to the mature protein, i.e. beginning [EEAS...].
