# Supplementary material for: Dynamic instability of the major urinary protein gene family revealed by genomic and phenotypic comparisons between C57 and 129 strain mice
Source: Genome Biol. 2008 May 28;9(5):R91. doi: 10.1186/gb-2008-9-5-r91 (PMC2441477; doi:10.1186/gb-2008-9-5-r91)
Supplement: Additional data file 2 — The point of alignment inversion is seen to correspond to the location of a murine ERV. [file gb-2008-9-5-r91-S2.pdf]

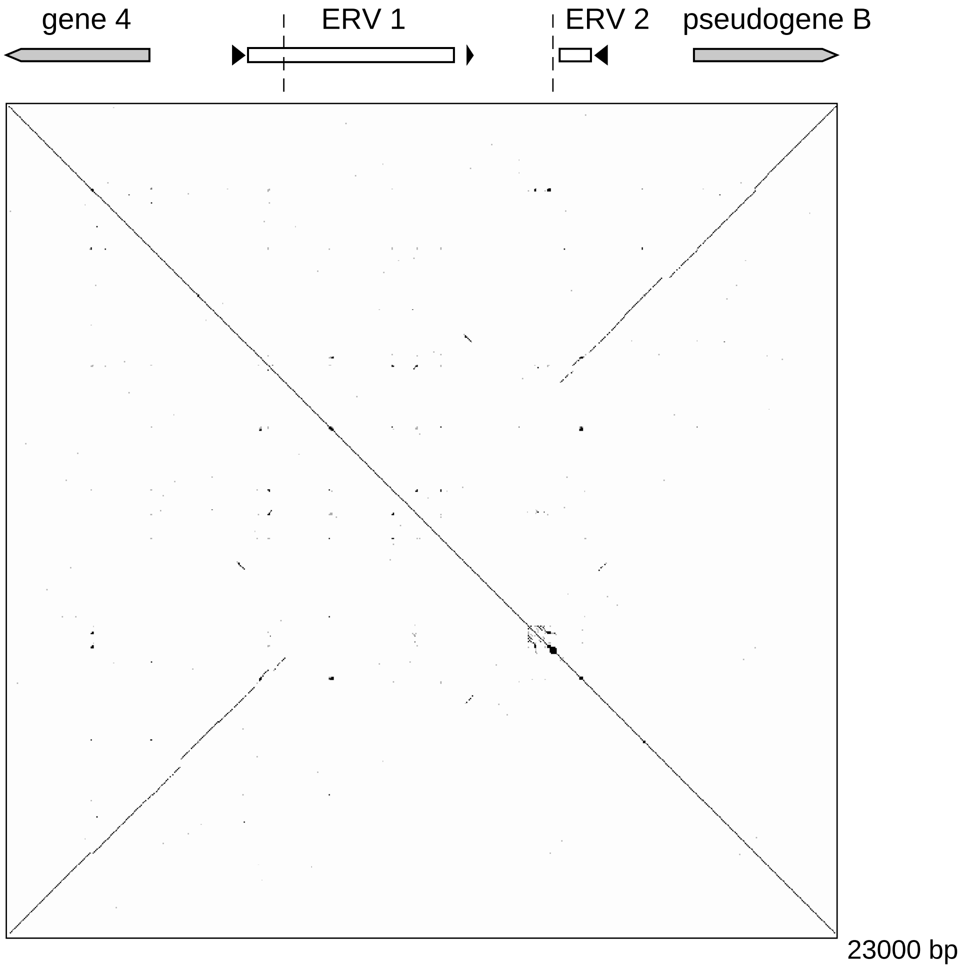

Additional data file 2. Dot-plot self comparison of genomic sequence between B6 gene 4 and pseudogene B. The genomic section runs between the 3‘ ends of these loci, which are represented as greyed polygons. The point of inversion is seen to correspond to the location of a murine ERV; the long terminal repeats (LTRs) are show as black triangles and the internal content as unfilled polygons. An intact element consists of a pair of LTRs in the same orientation flanking the central portion. Here, the architecture contains a largely intact element (ERV1) in head-to-head opposition to an adjacent fragment (ERV2). The dotted lines represent the point of fragmentation within this distal fragment compared against the intact element.
