# Supplementary material for: Dynamic instability of the major urinary protein gene family revealed by genomic and phenotypic comparisons between C57 and 129 strain mice
Source: Genome Biol. 2008 May 28;9(5):R91. doi: 10.1186/gb-2008-9-5-r91 (PMC2441477; doi:10.1186/gb-2008-9-5-r91)
Supplement: Additional data file 3 — Dot-plot comparison of the mass 18,893-associated duplication from the B6 genome. [file gb-2008-9-5-r91-S3.pdf]

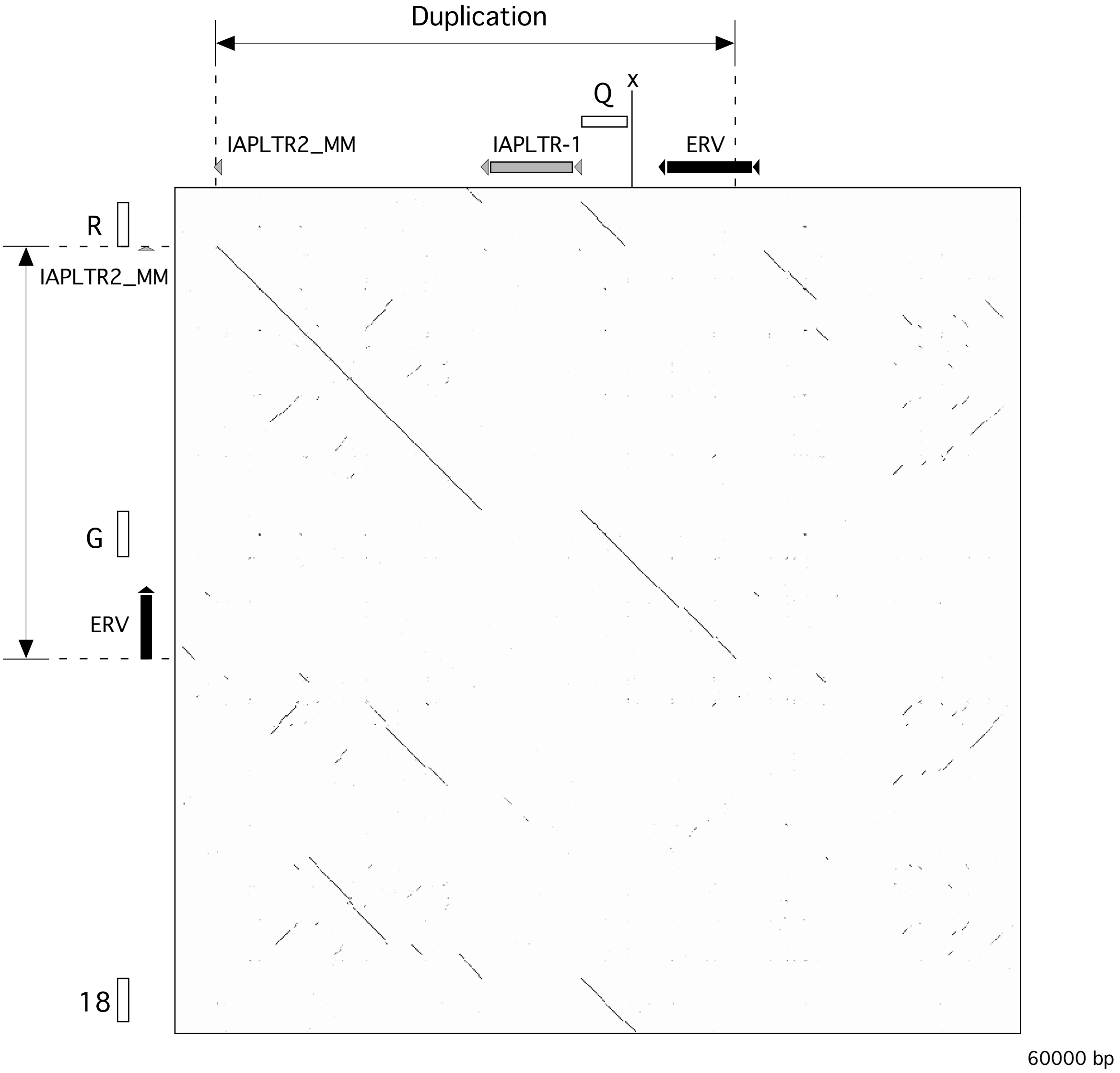

Additional data file 3. Dot-plot comparison of the mass 18893-associated duplication from the B6 genome. The proximal 60 kb of sequence flanking pseudogene Q is on the horizontal axis; the distal 60 kb flanking gene 17 on the vertical. Gene and pseudogenes are indicated as unfilled boxes, and labelled as in Figure 1. The duplication runs from 2.8 to 39.7 kb on the horizontal axis, interrupted by an intact IAPLTR-1 not present in the proximal sequence. The start and end points of the duplication are respectively located within the LTR of an IAPLTR2_MM retroelement (greyed triangle), lacking the central segment and flanking repeat, and the central portion of an ERV retroelement (black box with flanking triangles); breakpoints are indicated by dotted lines. Both of these elements are truncated on the horizontal sequence with respect to the vertical, indicating that the gene 17 region formed as a duplication from the pseudogene Q region. However, the marked event boundary at 33.3 kb represents an abrupt transition from 99.6% to 92.2% nucleotide similarity, suggesting this region has been subjected to more than one duplication event. Non-informative transposons and repeats have not been indicated.
