# Supplementary material for: Dynamic instability of the major urinary protein gene family revealed by genomic and phenotypic comparisons between C57 and 129 strain mice
Source: Genome Biol. 2008 May 28;9(5):R91. doi: 10.1186/gb-2008-9-5-r91 (PMC2441477; doi:10.1186/gb-2008-9-5-r91)
Supplement: Additional data file 4 — Details of the absence of MUP isoforms in the upper mass range of ESI-MS spectra of inbred mouse urine samples. [file gb-2008-9-5-r91-S4.ppt]

## Slide 1
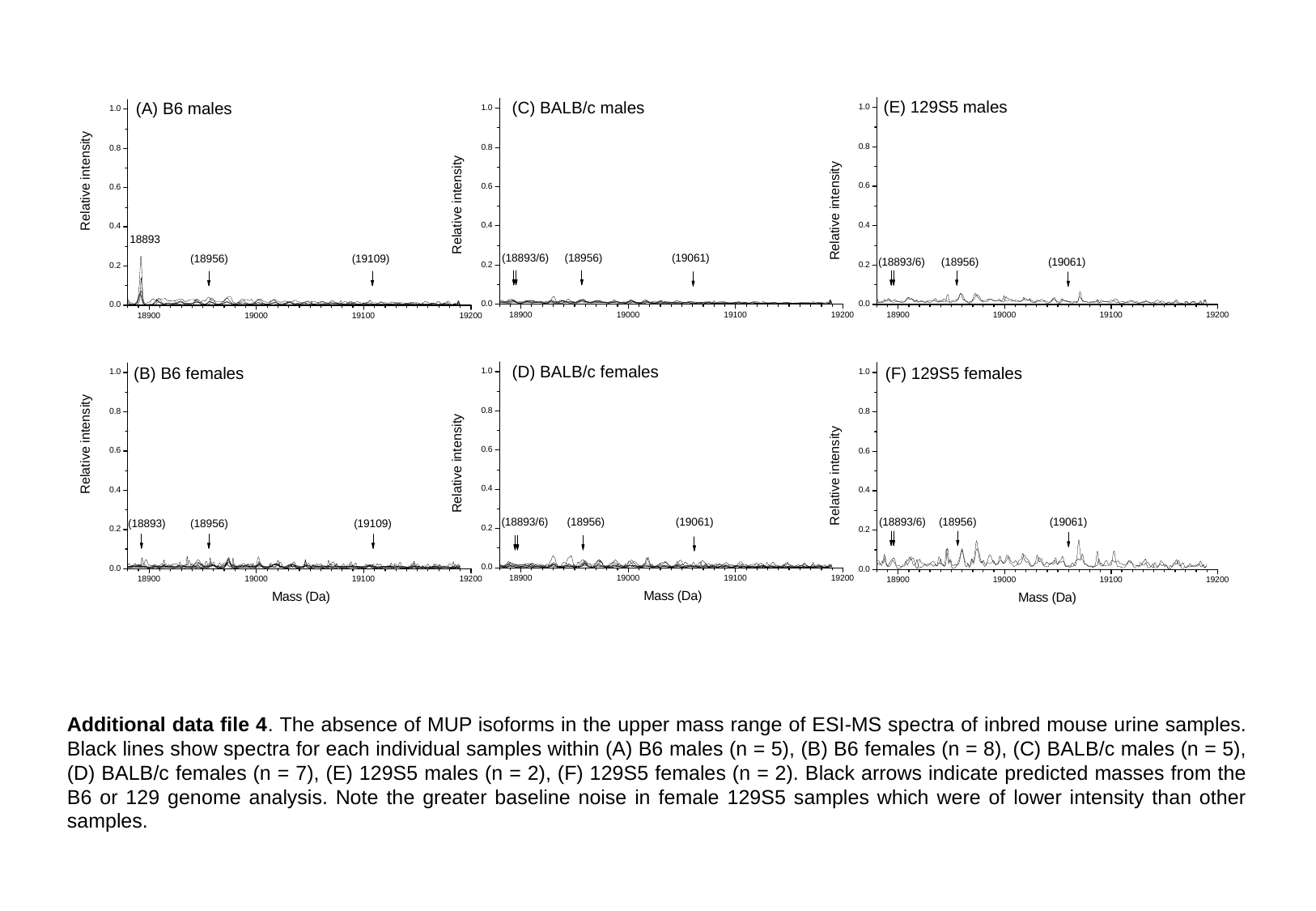

Additional data file 4. The absence of MUP isoforms in the upper mass range of ESI-MS spectra of inbred mouse urine samples. Black lines show spectra for each individual samples within (A) B6 males (n = 5), (B) B6 females (n = 8), (C) BALB/c males (n = 5), (D) BALB/c females (n = 7), (E) 129S5 males (n = 2), (F) 129S5 females (n = 2). Black arrows indicate predicted masses from the B6 or 129 genome analysis. Note the greater baseline noise in female 129S5 samples which were of lower intensity than other samples.
