# Supplementary material for: Dynamic instability of the major urinary protein gene family revealed by genomic and phenotypic comparisons between C57 and 129 strain mice
Source: Genome Biol. 2008 May 28;9(5):R91. doi: 10.1186/gb-2008-9-5-r91 (PMC2441477; doi:10.1186/gb-2008-9-5-r91)
Supplement: Additional data file 5 — Individual variation in ESI-MS mass spectra of MUP isoforms in urine. [file gb-2008-9-5-r91-S5.ppt]

## Slide 1
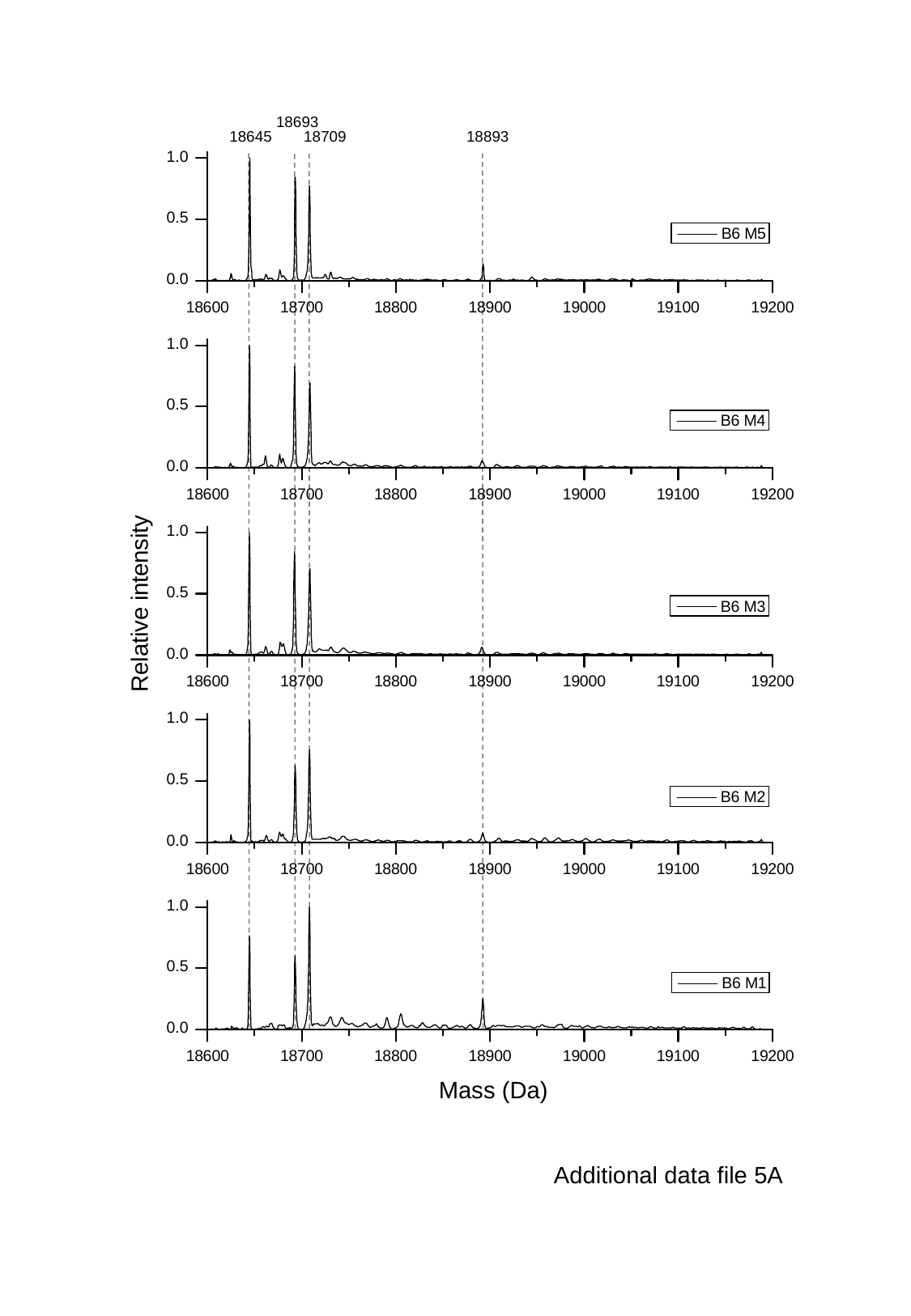

18693
18645
18709
18893
Additional data file 5A

## Slide 2
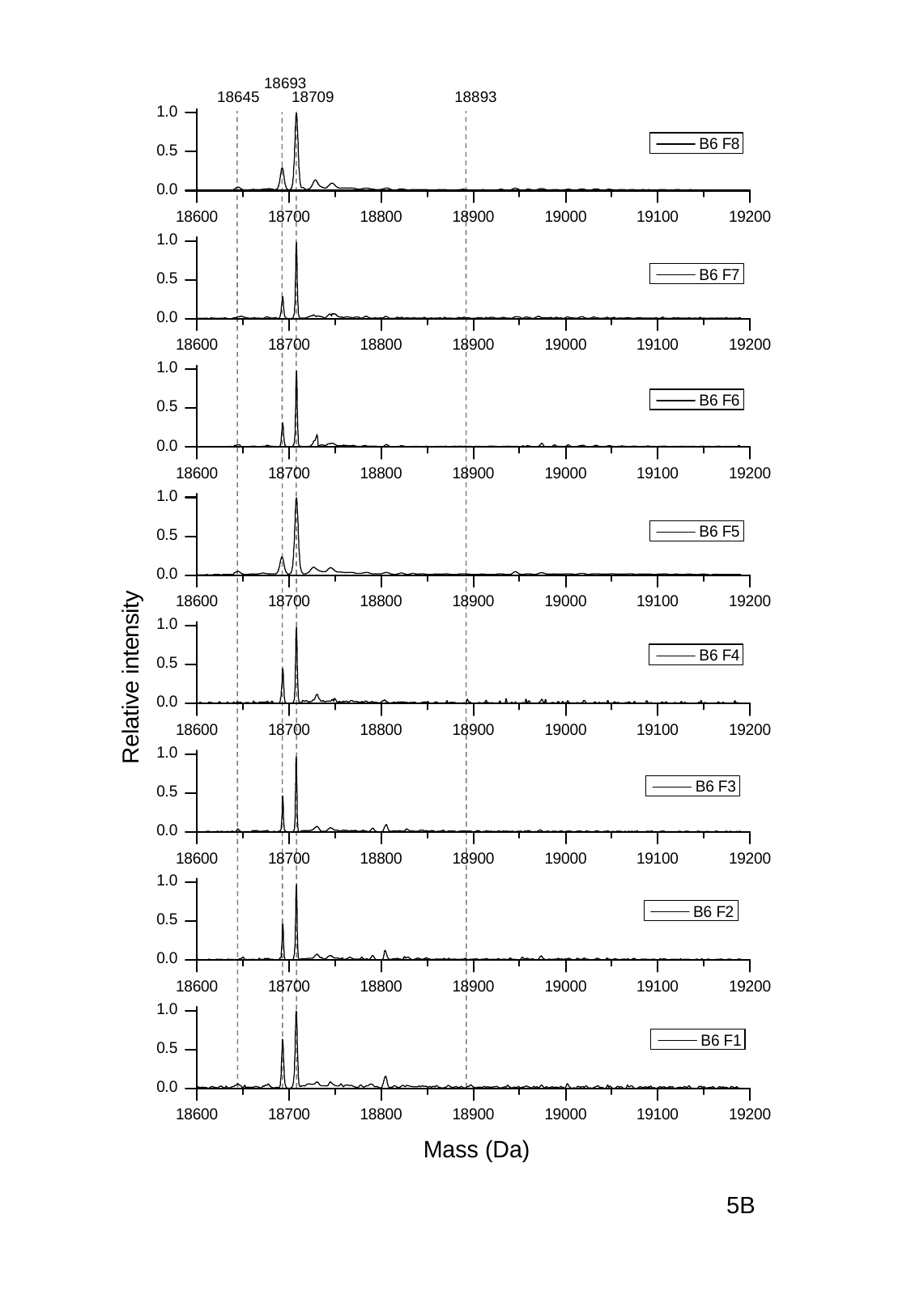

18693
18645
18709
18893
5B

## Slide 3
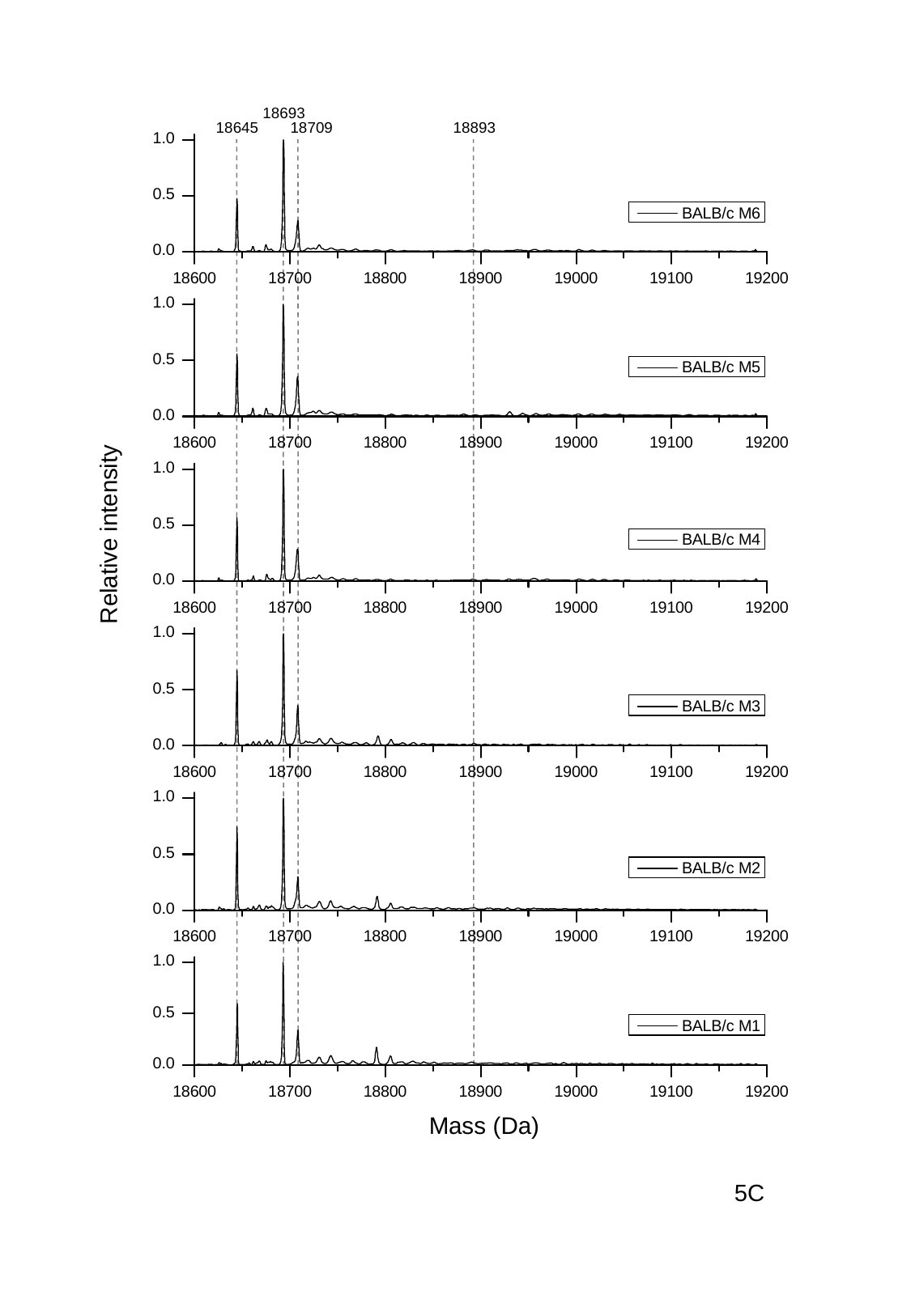

18693
18645
18709
18893
5C

## Slide 4
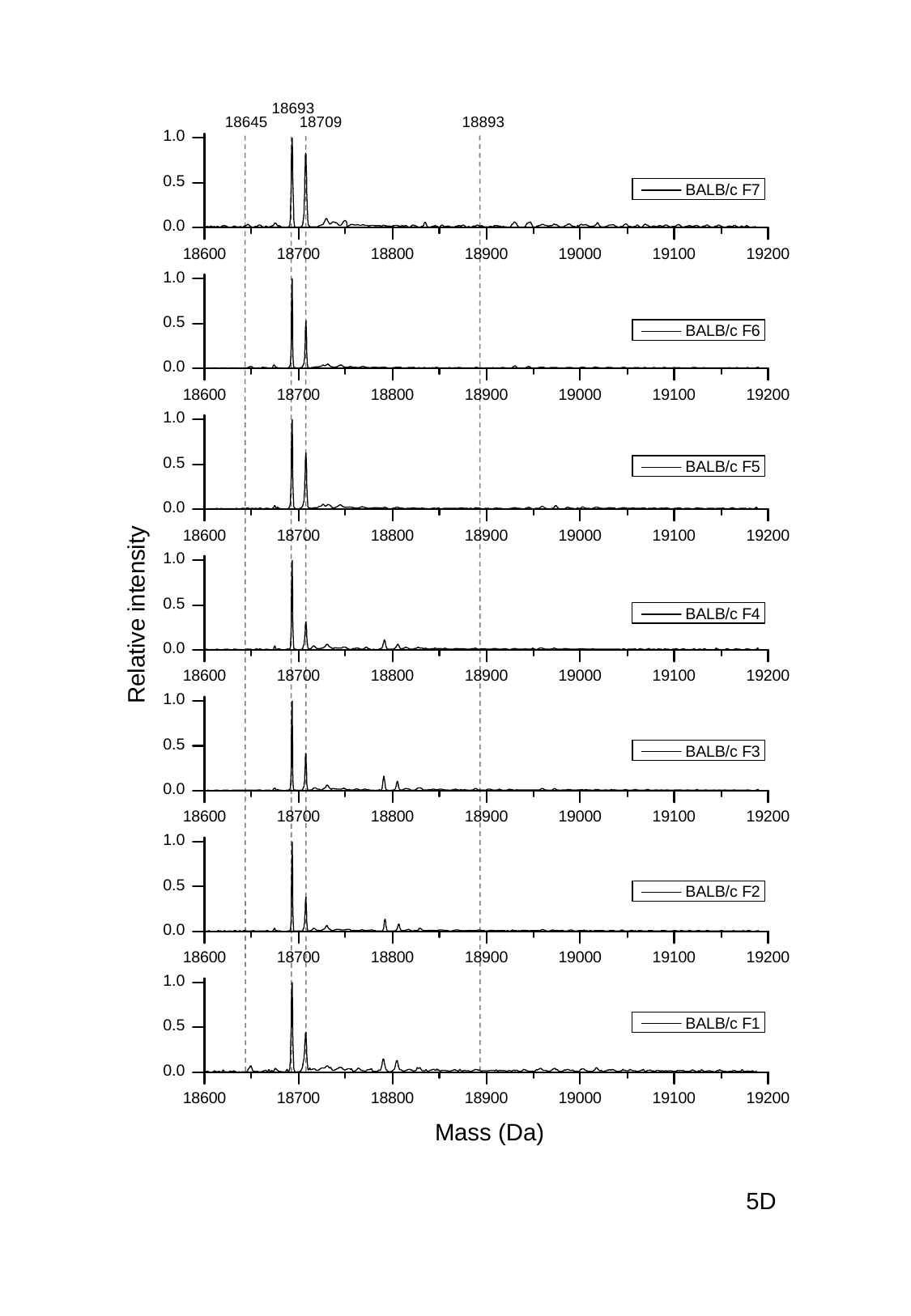

18693
18645
18709
18893
5D

## Slide 5
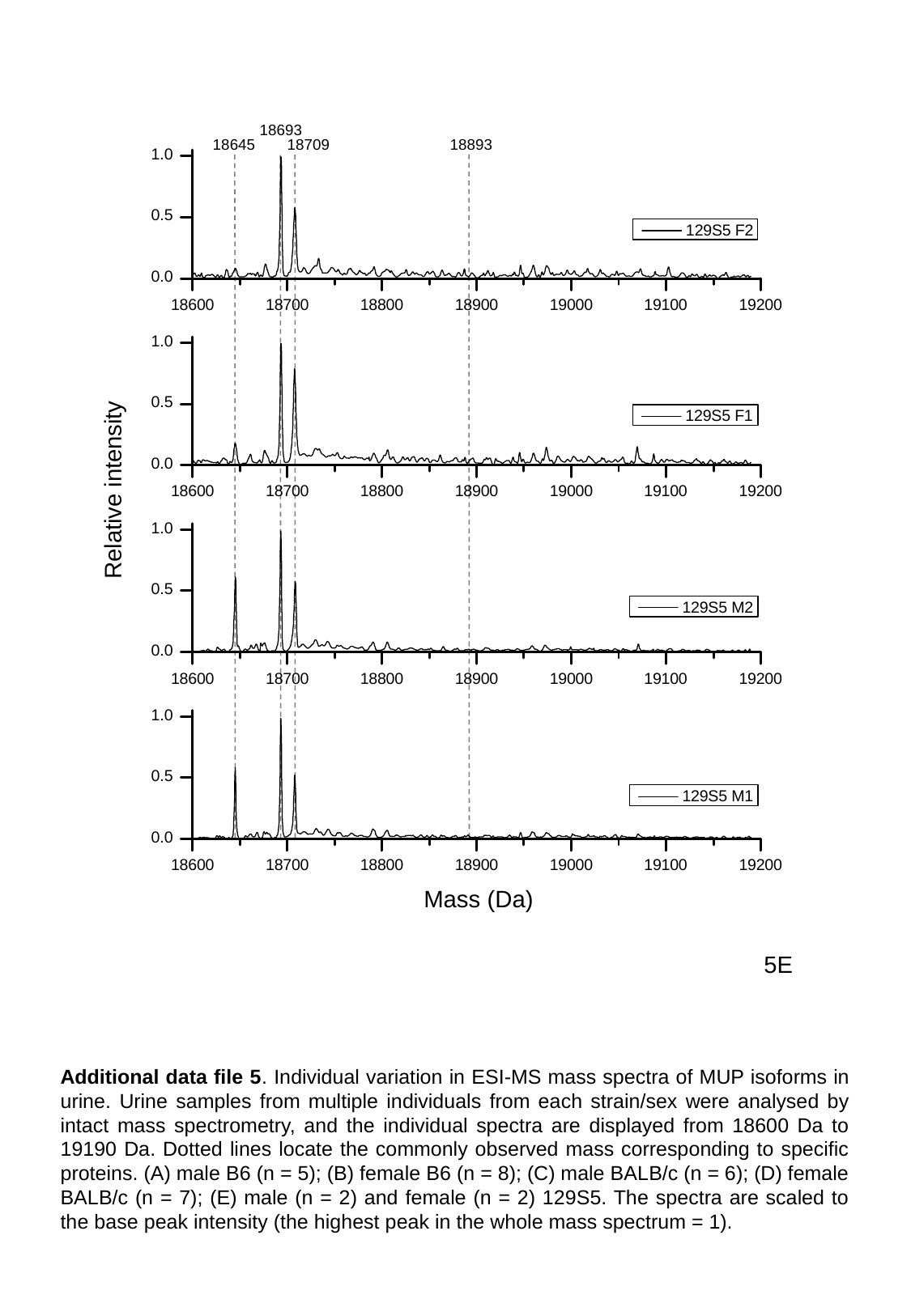

18693
18645
18709
18893
5E
Additional data file 5. Individual variation in ESI-MS mass spectra of MUP isoforms in urine. Urine samples from multiple individuals from each strain/sex were analysed by intact mass spectrometry, and the individual spectra are displayed from 18600 Da to 19190 Da. Dotted lines locate the commonly observed mass corresponding to specific proteins. (A) male B6 (n = 5); (B) female B6 (n = 8); (C) male BALB/c (n = 6); (D) female BALB/c (n = 7); (E) male (n = 2) and female (n = 2) 129S5. The spectra are scaled to the base peak intensity (the highest peak in the whole mass spectrum = 1).
