# Supplementary material for: Dynamic instability of the major urinary protein gene family revealed by genomic and phenotypic comparisons between C57 and 129 strain mice
Source: Genome Biol. 2008 May 28;9(5):R91. doi: 10.1186/gb-2008-9-5-r91 (PMC2441477; doi:10.1186/gb-2008-9-5-r91)
Supplement: Additional data file 6 — Increasing the volume of urine loaded onto a native PAGE gel does not change the banding pattern observed once the essential banding pattern has become visible. [file gb-2008-9-5-r91-S6.ppt]

## Slide 1
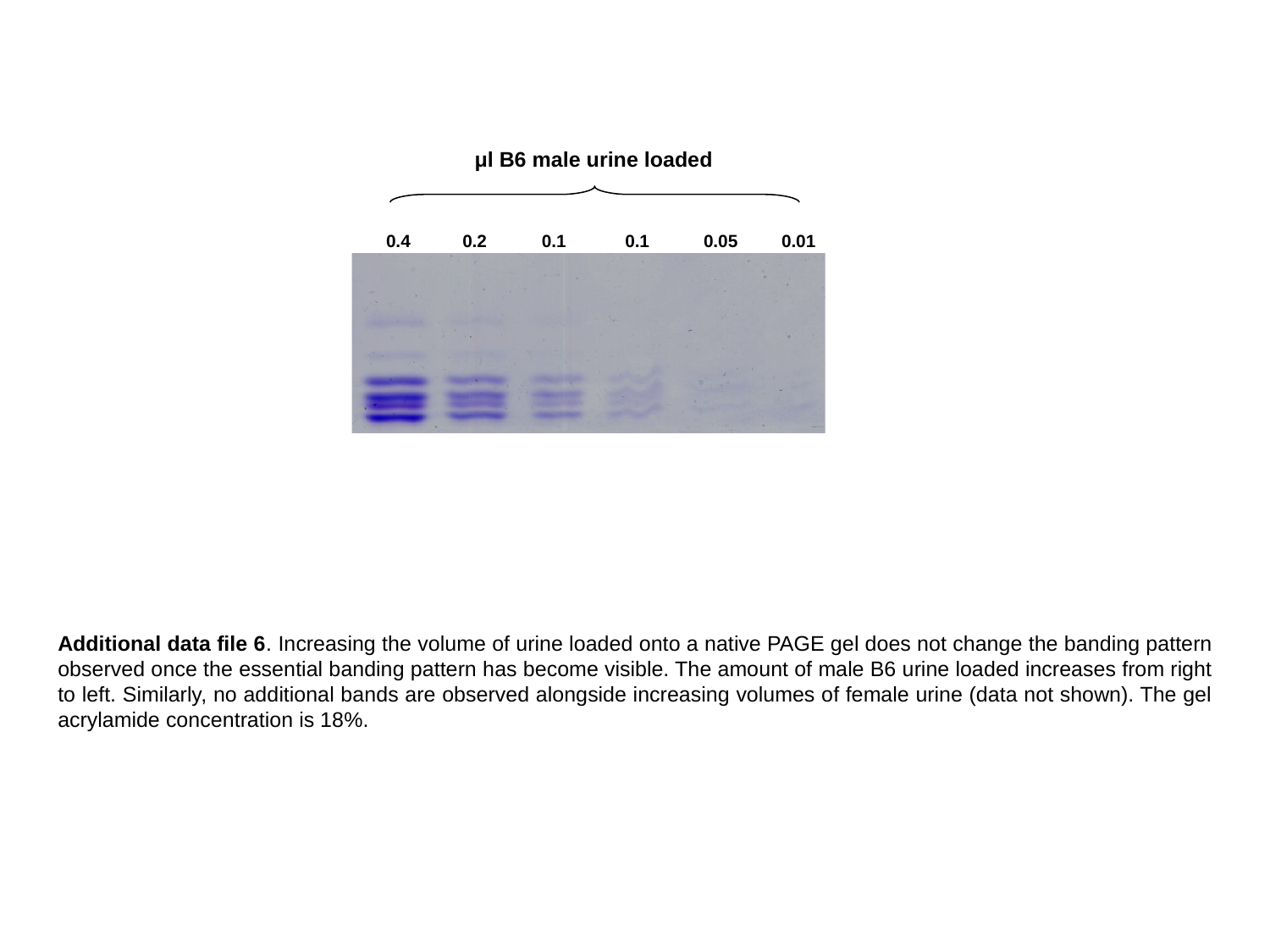

μl B6 male urine loaded
0.4
0.2
0.1
0.1
0.05
0.01
Additional data file 6. Increasing the volume of urine loaded onto a native PAGE gel does not change the banding pattern observed once the essential banding pattern has become visible. The amount of male B6 urine loaded increases from right to left. Similarly, no additional bands are observed alongside increasing volumes of female urine (data not shown). The gel acrylamide concentration is 18%.
